# Characterising airway obstructive, dysanaptic and PRISm phenotypes of prematurity-associated lung disease

**DOI:** 10.1136/thorax-2022-219301

**Published:** 2023-02-01

**Authors:** Michael Cousins, Kylie Hart, Sarah J Kotecha, A John Henderson, W John Watkins, Andrew Bush, Sailesh Kotecha

**Affiliations:** 1 Department of Child Health, Cardiff University School of Medicine, Cardiff, UK; 2 MRC Integrative Epidemiology Unit, Population Health Sciences, Bristol Medical School, University of Bristol, Bristol, UK; 3 Centre for Paediatrics and Child Health, Imperial College of Medicine, London, UK

**Keywords:** paediatric lung disaese

## Abstract

**Introduction:**

Although obstructive airway disease has been shown to be associated with prematurity, other spirometry phenotypes are less well described.

**Objectives:**

We characterised abnormal spirometry phenotypes in preterm-born children, including prematurity-associated obstructive lung disease (POLD, forced expiratory volume in 1 s (FEV_1_)<lower limit of normal (LLN), FEV_1_/forced vital capacity (FVC)<LLN), prematurity-associated preserved ratio of impaired spirometry (pPRISm, FEV_1_<LLN, FEV_1_/FVC≥LLN) and prematurity-associated dysanapsis (pDysanapsis, FEV_1_≥LLN, FEV_1_/FVC<LLN), and associated them with early life factors, bronchodilator responsiveness and fractional exhaled nitric oxide (FE_NO_).

**Methods:**

768 children, aged 7–12 years, underwent FE_NO_ measurements and spirometry before and after salbutamol. Groups were compared using parametric tests; multinomial regression was used.

**Results:**

22.6% of 544 preterm-born (mean gestation: 31 weeks) and 9.2% of 195 term-born children, with satisfactory data available, were classified into one of four abnormal spirometry groups. Each phenotype was generally more prevalent in preterm-born children than in the term-born children. For the preterm group, POLD-reversible (4.4%) was associated with increased FE_NO_, bronchopulmonary dysplasia (BPD) and intrauterine growth restriction. POLD-fixed group (3.3%) did not have increased FE_NO_ but was associated with BPD. 41% of the pDysanapsis group (5.9%) had bronchodilator response, 31% had increased FE_NO_ and was associated with postnatal weight gain. In the pPRISm group (9%), 13% responded to bronchodilators, FE_NO_ was not increased and was non-significantly associated with body mass index (p=0.064).

**Conclusions:**

Further to airway obstruction, we describe airway dysanapsis and pPRISm spirometry phenotypes in survivors of prematurity, both of which have poor outlook in other disease groups. By identifying specific phenotypes, targeted therapy can be developed to improve long-term outcomes.

**WHAT IS ALREADY KNOWN ON THIS TOPIC**

- Obstructive lung disease following preterm birth has been previously described; however, little is known about other potential patterns or phenotypes of lung disease in such children.

**WHAT THIS STUDY ADDS**

- This study characterises four groups of preterm-born children with evidence of lung dysfunction, each with differential associations with spirometry, exhaled nitric oxide and reversibility, as well as early and current life factors.

**HOW THIS STUDY MIGHT AFFECT RESEARCH, PRACTICE OR POLICY**

- Using the clinical phenotypes we have identified, spirometry measurements can be used to identify children who can benefit from treatment to improve lung dysfunction, and also identify children who need long-term surveillance due to later comorbidities associated with their pattern of lung disease.

## Introduction

Low lung function observed in prematurity-associated lung disease (PLD),^1–3^ including those who develop bronchopulmonary dysplasia (BPD, also known as chronic lung disease of prematurity), is now considered a precursor for early onset chronic obstructive pulmonary disease (COPD).^4^ Although historical focus has been on preterm-born survivors who had BPD in infancy,^5 6^ it is increasingly recognised that late-preterm-born children (especially those born at 33–36 weeks’ gestation) also have lung function decrements.^1 7^ We have recently reported that gestational age at birth and intrauterine growth restriction (IUGR) are better predictors of low lung function in childhood than BPD in multivariable models.^8^ Our systematic review showed improved spirometry after acute administration of bronchodilators, but the specific group of preterm-born children with PLD that would benefit the most is unclear.^9^ Furthermore, fractional exhaled nitric oxide (FE_NO_) has not shown to be increased in children who had BPD in infancy.^10^ In contrast, our recent data showed that FE_NO_ was decreased by inhaled corticosteroids (ICS) either alone or in combination with long-acting beta_2_ receptor agonists (LABA) but not after placebo treatment in a randomised controlled trial suggesting that some children with PLD may have eosinophilic airway disease.^11^

However, while airway obstruction has been described after preterm birth, little is known about other patterns of abnormal spirometry of PLD, including preserved ratio of impaired spirometry (PRISm) and dysanaptic airway growth. PRISm is an important phenotype as it is associated with increased risk of developing COPD and cardiovascular disease and with all-cause mortality in middle-aged and old-aged adult populations,^12 13^ but has yet to be evaluated in children including in the preterm population; and dysanaptic airway growth is associated with worse asthma.^14^

We hypothesised that there are multiple spirometry phenotypes in PLD, including:

- prematurity-associated obstructive lung disease that is bronchodilator responsive (POLD-reversible),
- persistent airflow limitation of prematurity that does not respond to bronchodilators (POLD-fixed),
- prematurity-associated PRISm (pPRISm),
- dysanapsis of prematurity (pDysanapsis).

We also examined early and current life factors which were associated with each phenotype, in order to begin to understand how to personalise preventative strategies.

## Methods

### Study population

The Respiratory Health Outcomes in Neonates (RHiNO, EudraCT: 2015-003712-20) study has been described previously.^8 11^ Briefly, children from a previous questionnaire study^1 15^ were supplemented with additional preterm-born children, and were mailed a respiratory questionnaire for their parents to complete. Responders to the questionnaire were invited to join RHiNO for a home or hospital visit to obtain anthropometric details, perinatal and respiratory history (supplemented by examination of the child’s medical records), measurement of FE_NO_ (NIOX VERO, Circassia, Oxford, UK) and spirometry (Microloop, CareFusion, Wokingham, UK) before and after administration of salbutamol (400 μg administered via a spacer device) as described in the online supplemental file. Spirometry was performed and quality controlled according to the American Thoracic Society/European Respiratory Society guidelines,^16^ and per cent predicted and z-scores were calculated using Global Lung Initiative reference equations.^17^

10.1136/thorax-2022-219301.supp1 Supplementary data

Children aged 7–12 years, born at ≤34 weeks’ gestation for the preterm group or at ≥37 weeks’ gestation for the term control group were invited. Children born at 35–36 weeks’ gestation were not included as they have previously been shown to have lesser degrees of lung function deficits.^7^ Recruitment took place prospectively between November 2016 and September 2019. A subgroup underwent extended assessments including skin prick testing (see online supplemental file). Children with significant congenital, cardiac or neurodevelopmental abnormalities were excluded. Z-scores for birth weight, current weight, height and BMI and IUGR (defined as <10th centile for birth weight adjusted for sex and gestation) were calculated using the LMS growth program.^18^ Deprivation scores and quintiles were estimated from the participants’ postcodes using the Welsh Index of Multiple Deprivation score, which is a validated measure of deprivation based on eight domains including wealth, schooling and home ownership.^19^ BPD was defined as supplemental oxygen-dependency of 28 days of age or greater for those born <32 weeks’ gestation and at 56 days of age for those ≥32 weeks’ gestation).^20^

### Definitions of spirometry phenotypes

POLD in the preterm group was defined as FEV_1_ and FEV_1_/FVC ratio less than the lower limit of normal (LLN) and further subdivided into reversible (POLD-reversible) and fixed airway (POLD-fixed) disease if their FEV_1_ improved by 10% or not, respectively, after bronchodilator administration. Dysanapsis was defined according to the American Thoracic Society/European Respiratory Society (ATS/ERS) definition as FEV_1_≥LLN and FEV_1_/FVC<LLN^21^ and pPRISm as FEV_1_<LLN and FEV_1_/FVC ratio≥LLN.^11 12^ Equivalent phenotypes were defined similarly for the term group. Preterm-born and term-born children with FEV_1_≥LLN and FEV_1_/FVC ratio≥LLN were considered as control groups. These definitions and other abbreviations are described in table 1.

**Table 1:** Abbreviations and definitions for grouping participants based on lung function

Abbreviation	Definition
PLD	Prematurity-associated lung disease
POLD-reversible	Prematurity-associated obstructive lung disease with evidence of bronchodilator reversibility (FEV_1_<LLN; FEV_1_/FVC ratio<LLN; bronchodilator response ≥10%)
POLD-fixed	Prematurity-associated obstructive lung disease with NO bronchodilator reversibility (FEV_1_<LLN; FEV_1_/FVC ratio<LLN; bronchodilator response <10%)
pPRISm	Prematurity-associated preserved ratio of impaired spirometry (FEV_1_<LLN; FEV_1_/FVC ratio≥LLN)
pDysanapsis	Prematurity-associated dysanapsis (FEV_1_≥LLN; FEV_1_/FVC<LLN)
Preterm controls	FEV_1_≥LLN; FEV_1_/FVC ratio≥LLN
Term controls	FEV_1_≥LLN; FEV_1_/FVC ratio≥LLN

FEV_1_, forced expiratory volume in 1 s; FVC, forced vital capacity; LLN, lower limit of normal.

### Statistical analyses

Data are given as means and SD, or medians and ranges, as appropriate. One-way analysis of variance with Bonferroni correction were used to compare means as appropriate. The χ^2^ test was used to compare independence of categorical variables. ORs (and 95% CIs) were calculated to compare each phenotype in the preterm group against the phenotype in the term group. Multinomial regression was used to identify early and current life factors that were associated with spirometry phenotypes. Data were analysed using SPSS V.26 (IBM, New York, USA). P value <0.05 was considered statistically significant.

## Results

From 1426 returned questionnaires, 768, including 565 preterm-born and 203 term-born, children participated in a home or hospital visit. As previously reported, lower gestational age and birth weight; and fewer of the most-deprived population were noted for responders (31.0 weeks, 1703 g) and non-responders (31.6 weeks, 1828 g) to the questionnaires.^8^ Twenty-one preterm-born and 8 term-born children’s spirometry did not pass quality control criteria thus spirometry data were available from 544 preterm-born and 195 term-born children (table 2). Two hundred seventy-six children were born at <32 weeks’ gestation including 99 born at 28 weeks or less. One hundred eight children had mild/moderate/severe BPD diagnosed in infancy.

**Table 2:** Participant demographics by phenotypes

Characteristic	POLD-reversible	POLD-fixed	pPRISm	pDysanapsis	Preterm—normal spirometry (PTc)	Term
Number	24	18	49	32	421	195
Per cent of preterm population	4.4%	3.3%	9.0%	5.9%	77.4%	–
Male	9 (38%)	9 (50%)	28 (57%)	17 (53%)	216 (51%)	100 (51%)
Caucasian ethnicity	21 (88%)	17 (94%)	49 (100%)	30 (94%)	398 (95%)	188 (96%)
Current age, years (SD)	9.6 (9.6)	10.0 (10)	10.3 (1.3)*†‡	9.4 (9.4)	9.6 (1.3)	9.7 (1.2)
Current height, cm (SD)	137.7 (10.0)	140.4 (10.4)	143.5 (10.3)	138.7 (10.2)	139.5 (10.4)	142.0 (9.3)
Current height, z-score (SD)	0.243 (1.19)	0.315 (0.96)	0.551 (1.36)	0.544 (1.06)	0.580 (1.14)*	0.889 (1.09)
Current weight, kg (SD)	33.6 (11.7)	34.4 (9.2)	36.4 (10.4)	36.6 (11.9)	35.2 (10.6)	37.0 (10.4)
Current weight, z-score (SD)	0.090 (1.46)	0.226 (1.02)	0.237 (1.42)	0.729 (1.22)	0.512 (1.12)	0.744 (1.07)
Current body mass index, kg/m^2^ (SD)	17.3 (3.9)	17.2 (2.4)	17.4 (3.2)	18.6 (4.1)	17.7 (3.3)	18.1 (3.3)
Current body mass index, z-score (SD)	−0.048 (1.50)	0.070 (1.16)	−0.039 (1.35)	0.627 (1.34)	0.311 (1.21)	0.438 (1.18)
z-score change from birth to current weight (SD)	0.458 (1.515)	0.232 (1.370)	0.410 (1.717)	0.919 (1.252)	**0.240 (1.497**)**	0.682 (1.226)
Median gestational age, weeks, (range)	30 (24, 34)***	29.5 (24, 34)***	32 (25, 34)***	32.5 (23, 34)***	32 (23, 34)***	40 (37, 42)
Median birth weight, g (range)	1120 (550, 2353)***	1349 (625, 2608)***	1644 (500, 2750)***	1691 (620, 2810)***	1757 (450, 3912)***	3430 (2155, 4916)
Birth weight, z-score (SD)	−0.368 (1.48)	−0.006 (1.18)	−0.173 (1.24)	−0.19 (1.17)	0.272 (1.36)	0.062 (0.97)
Intrauterine growth restriction	**9 (38%)** ^**§‡‡‡**^ ***	4 (22%)**	7 (14%)*	7 (22%)***	49 (12%)**	9 (5%)
Antenatal steroids	21 (88%)***	16 (94%)***	44 (90%)***	27 (96%)***	343 (87%)***	4 (2%)
Bronchopulmonary dysplasia	**10 (42%)** ^**†‡‡**^ ***	**8 (44%)** ^**†‡‡**^ ***	12 (25%)***	4 (13%)***	74 (18%)***	0 (0%)
Wheeze ever	**19 (79%)** ^**§‡‡**^ ***	**13 (72%)** ^**‡**^ ***	25 (51%)**	23 (72%)^‡‡^***	201 (48%)***	51 (26%)
Recent wheeze (<12 months)	14 (58%)^§§‡‡‡^***	7 (39%)**	11 (22%)	18 (56%)^§§‡‡‡^***	102 (24%)**	25 (13%)
Doctor diagnosed asthma	9 (38%)^‡‡‡^***	**6** (**33%**)^‡^ ***	10 (20%)**	11 (36%)^‡‡‡^***	52 (12%)**	10 (5%)
Inhalers last 12 months	**10 (42%)** ^**§‡‡‡**^ ***	**6 (33%)** ^‡^ ***	8 (16%)*	**13 (41%)** ^§^ ^‡‡‡^***	56 (13%)**	12 (6%)
Current preventer inhaler	7 (29%)^‡‡^***	**5 (28%)** ^‡^ ***	7 (14%)**	9 (28%)^‡‡^***	39 (9%)*	8 (4%)
Maternal smoking in pregnancy	2 (8%)	2 (11%)	5 (10%)	5 (17%)*	48 (12%)*	11 (6%)
Current maternal smoking	4 (17%)*	1 (6%)	5 (10%)	6 (19%)**	58 (14%)***	8 (4%)
FEV_1_, %predicted (SD)	63.8 (10.2)^§§§†††‡‡‡^***	69.7 (10.6)^†††‡‡‡^***	75.8 (4.5)^†††‡‡‡^***	**89.2 (6.1**)^**‡‡‡**^ ***	95.6 (8.9)	95.7 (10.2)
FEV_1_, z-score (SD)	**−3.04 (0.81)** ^**§§§†††‡‡‡**^ ***	**−2.58 (0.89)** ^**†††‡‡‡**^ ***	**−2.07 (0.37)** ^**†††‡‡‡**^ ***	**−0.94 (0.53)** ^**‡‡**^ **	−0.38 (0.77)	−0.38 (0.87)
FEF_25%–75%_, %predicted (SD)	**36.2 (10.6)** ^**§§§†††‡‡‡**^ ***	**41.6 (10.0)** ^**§§§‡‡‡**^ ***	**63.1 (10.2)** ^**‡‡‡**^ ***	**54.7 (6.4)** ^**‡‡**^ **	84.1 (16.3)	86.4 (19.3)
FEF_25%–75%_, z-score (SD)	−3.24 (0.64)^§§§†††‡‡‡***^	**−3.05 (0.79)** ^**§§§††‡‡‡**^ ***	**−1.8 (0.54)** ^**‡‡‡**^ ***	**−2.2 (0.36)** ^**‡‡**^ **	−0.75 (0.75)	−0.66 (0.91)
FVC, %predicted (SD)	82.8 (9.0)^†††‡‡‡^***	85.8 (9.1)^†††‡‡‡^***	77.6 (6.0)^†††¶‡‡‡^***	106.1 (8.0)	**96.4 (9.6**)^**†††**^	**96.2 (10.3)^**†††**^**
FVC, z-score (SD)	**−1.51 (0.80)** ^**†††‡‡‡**^ ***	**−1.22 (0.77)** ^**†††‡‡‡**^ ***	**−1.94 (0.52)** ^†††¶‡‡‡***^	0.51 (0.67)	**−0.32 (0.82)** ^**†††**^	**−0.33 (0.88)^**†††**^**
FEV_1_/FVC, ratio (SD)	0.68 (0.08)^§§§††‡‡‡^***	0.71 (0.07)^§§§‡‡‡^***	0.85 (0.06)	**0.74 (0.03**)^**§§§‡‡‡**^ ***	0.87 (0.05)	0.87 (0.06)
FEV_1_/FVC, z-score (SD)	**−2.58 (0.63)** ^**§§§‡‡‡**^ ***	**−2.35 (0.56)** ^**§§§‡‡‡**^ ***	−0.33 (0.90)	**−2.06 (0.29**)^**§§§‡‡‡**^ ***	−0.12 (0.90)	−0.07 (0.98)

Data are given as means and SDs for continuous variables, or number and percentage for categorical variables, unless specifically stated.

Significant vs ¶POLD – Fixed, §pPRISm, †pDysanapsis, ‡PT_c_, * T_c_ (single symbol denotes significance level <0.05, double symbol <0.01, triple symbol<0.001).

FEV_1_, forced expiratory volume in 1 s; FVC, forced vital capacity; pDysanapsis, dysanapsis of prematurity; POLD, prematurity-associated obstructive lung disease; pPRISm, prematurity-associated PRISm.

### Classification of preterm-born and term-born children into spirometry phenotypes

**Figure 1:**
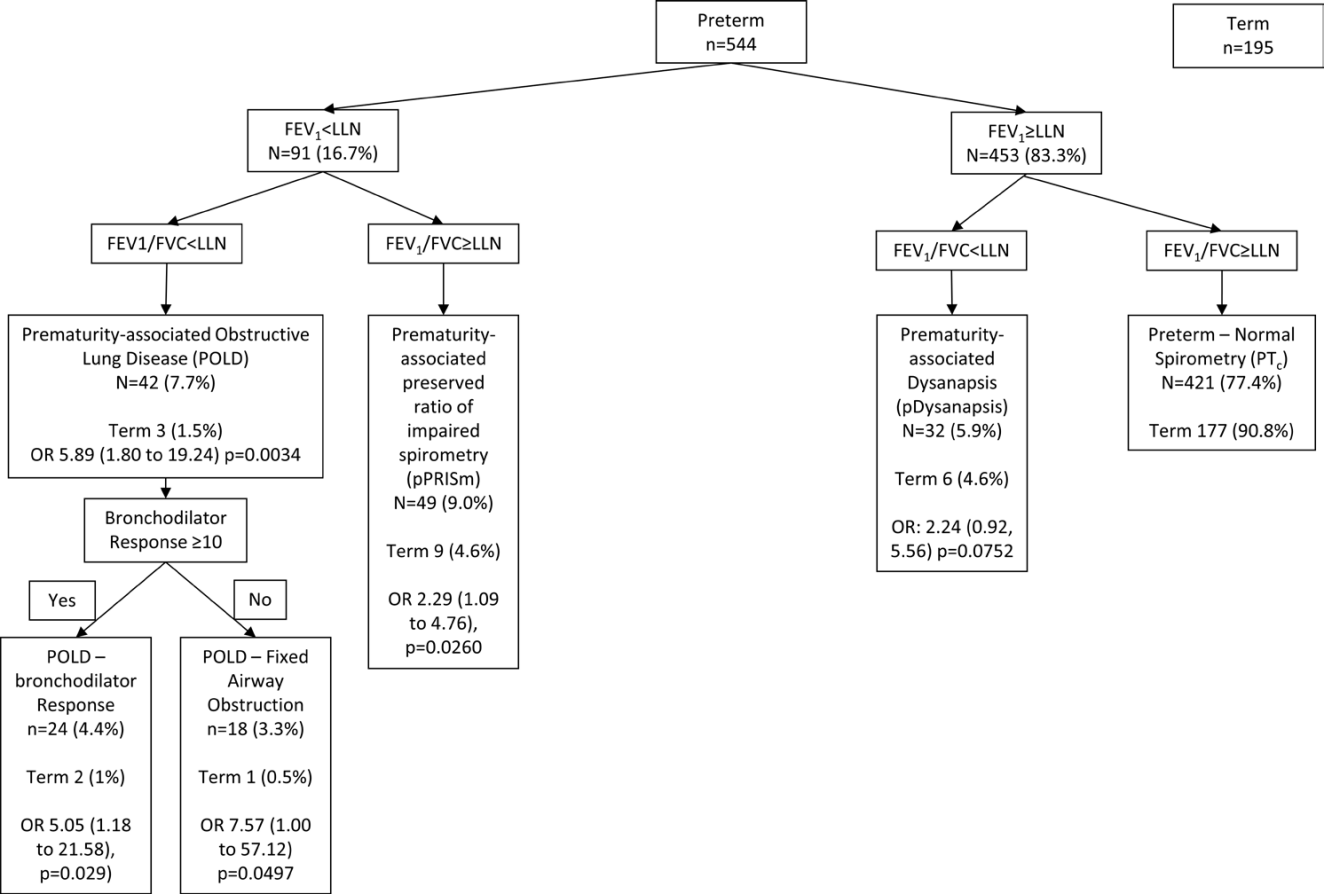
Classification of preterm and term groups based on their spirometry measures and bronchodilators responses. FEV_1_, forced expiratory volume in 1 s; FVC, forced vital capacity; LLN, lower limit of normal.

Overall, 123/544 (22.6%) preterm-born children compared with 18/195 (9.2%) of term-born children were classified into one of the four spirometry phenotypes giving OR and 95% CIs of 2.87 (1.70 to 4.86, p=0.0001) when the preterm and term groups were compared (figure 1). For the preterm group, 91 (16.7%) with FEV_1_<LLN were further classified into 42 (7.7%) with FEV_1_/FVC ratio<LLN into who had an obstructive phenotype (POLD) and into 49 (9.0%) with FEV_1_/FVC ratio≥LLN into pPRISm phenotype. The POLD group was further classified into those who had (POLD-reversible, n=24, 4.4%) or did not have (POLD-fixed, n=18, 3.3%) bronchodilator responsiveness. Preterm-born children with FEV_1_≥LLN and FEV_1_/FVC ratio<LLN were classified into the pDysanapsis group (n=32, 5.9%) and the remaining 421 (77.4%) with FEV_1_ and FEV_1_/FVC ratio≥LLN were classified as the preterm control group. For the term group, 2 (1%, OR; 95% CI 5.05; 1.18 to 21.58; p=0.029), 1 (0.5%, 7.57; 1.00 to 57.12, p=0.049), 9 (4.6%, 2.29; 1.09 to 4.76; p=0.026) and 6 (4.6%; 2.24; 0.92 to 5.56; p=0.075) were classified into reversible and fixed obstructive, pPRISm and dysanapsis groups, respectively. The OR and 95% CI compared each phenotype between the preterm and term groups.

### Participant demographics

When the six groups were compared (table 2), the pPRISm group were marginally older, but weight, height and BMI were similar between the groups. The POLD-reversible group had more IUGR. BPD was more prevalent in the two POLD groups regardless of bronchodilator response. The two POLD groups and the pDysanapsis group were associated with more respiratory symptoms, doctor-diagnosed asthma and inhaler use. Spirometry was lower in all phenotypes identified when compared with the term control group with the lowest measures noted in the two POLD groups. Preterm controls had very similar spirometry to the term control group, hence were used as reference population in multinomial regression analyses.

### Bronchodilator response

**Figure 2:**
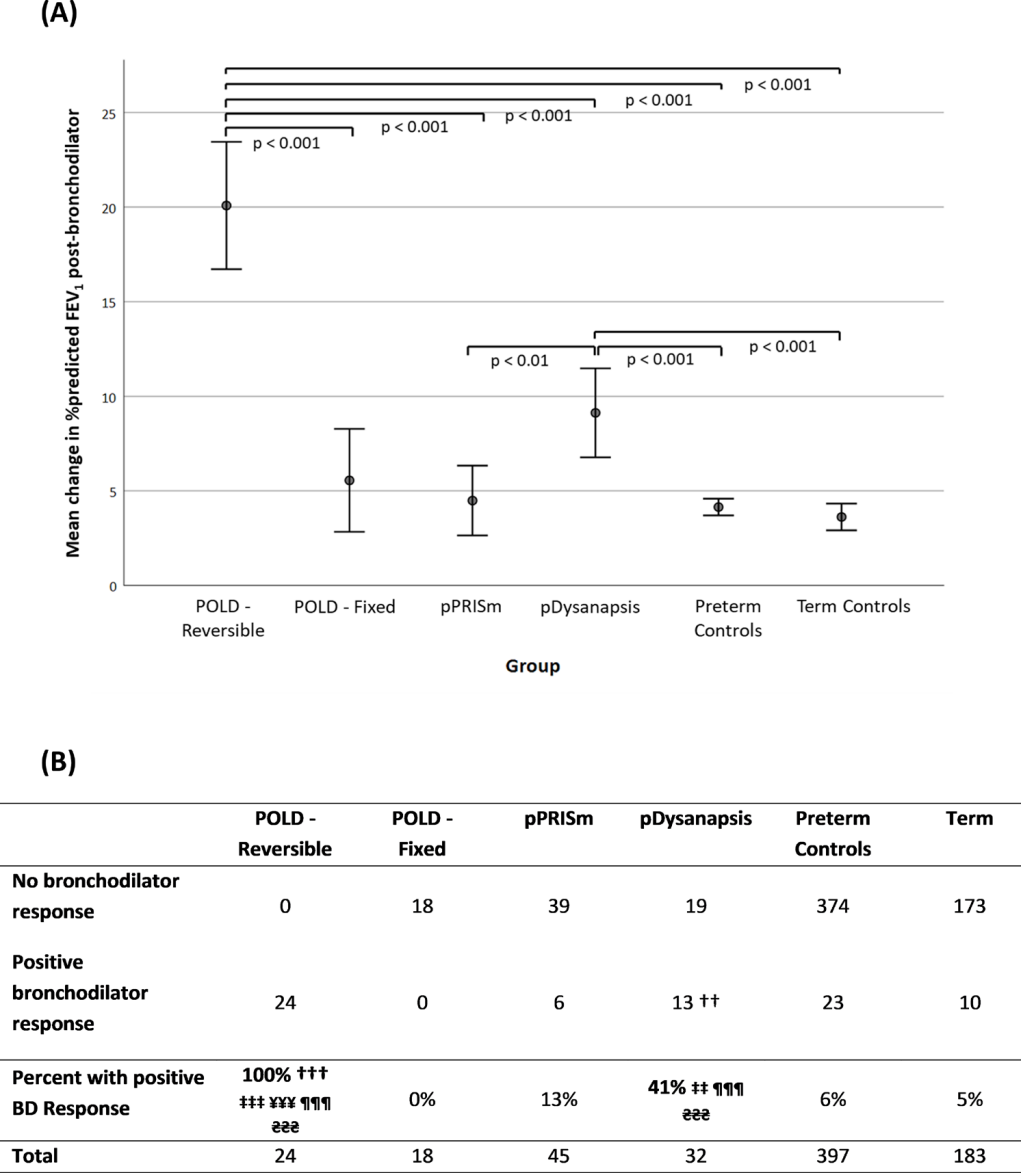
(A) Mean change in bronchodilator plotted against the different groups and (B) table showing the proportion of subjects with a positive bronchodilator response defined as 10% improvement in postbronchodilator %FEV_1_. Significant versus †POLD-fixed, ‡pPRISm, ¥pDysanapsis, ¶PT_c_, ₴T_c_ (single symbol denotes significance level <0.05, double symbol <0.01, triple symbol <0.001). FEV_1_, forced expiratory volume in 1 s; FVC, forced vital capacity; pDysanapsis, dysanapsis of prematurity; POLD, prematurity-associated obstructive lung disease; pPRISm, prematurity-associated PRISm.

Figure 2 shows the bronchodilator responses for the six groups. The largest and least increases were observed in the POLD-reversible group and POLD-fixed group (expected as defined on basis of bronchodilator response). FEV_1_ increased to ≥LLN after bronchodilator administration in 16/24 (67%) in the POLD-reversible group suggesting potential to reach spirometry within normal limits. Fewer of pPRISm (13%); and preterm (6%) or term (5%) control groups had a response but 41% of the pDysanapsis group responded to bronchodilators.

### FE_NO_ levels

**Figure 3:**
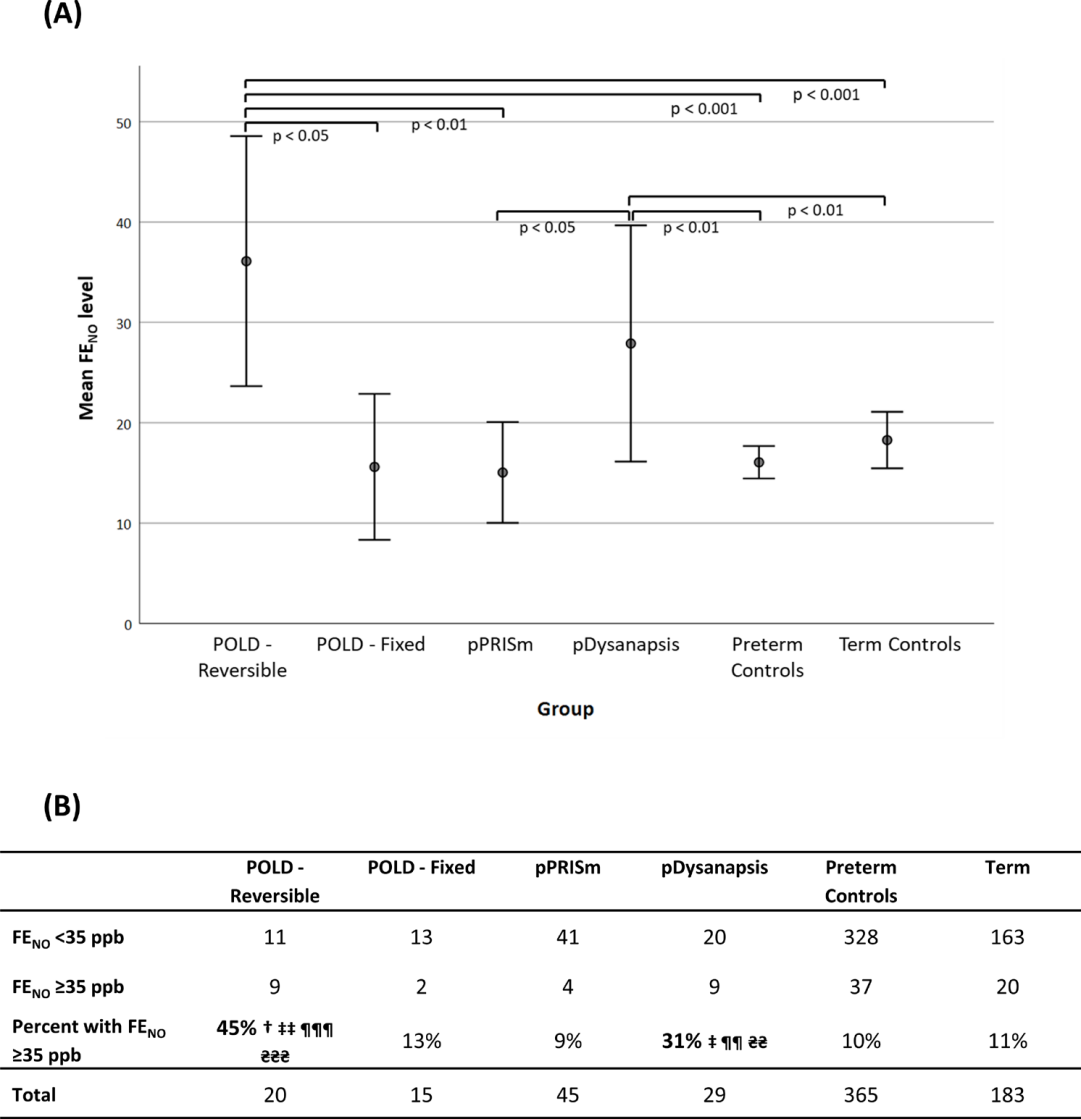
(A) Fractional exhaled nitric oxide (FE_NO_) levels plotted against the different groups and (B) table showing the proportion of subjects with FE_NO_>35 ppb. Significant versus *POLD-reversible, †PLD-fixed, ‡pPRISm, ¥pDysanapsis, ¶PT_c_, ₴T_c_ (single symbol denotes significance level <0.05, double symbol <0.01, triple symbol <0.001). pDysanapsis, dysanapsis of prematurity; POLD, prematurity-associated obstructive lung disease; pPRISm, prematurity-associated PRISm.

FE_NO_ levels were increased the most in the POLD-reversible group (mean: 36.1, 95% CI 23.7 to 48.6 ppb) and the pDysanapsis group (27.9; 16.1 to 39.7) but were similar in the pPRISm, POLD-fixed and control groups (figure 3); 45% and 31%, respectively of the POLD-reversible and pDysanapsis groups had FE_NO_ greater than the National Institute for Health and Care Excellence and ATS recommended 35 ppb, but this proportion was 13% or less in the remaining groups.^22 23^

### Skin prick testing

A subgroup of 237/739 (32%) children underwent skin prick testing (table 3). Eleven per cent of the term group and 20%–23% of the POLD-fixed, pPRISm and preterm controls had positive tests. Although the POLD-reversible group had greater positivity than the term groups (33% vs 11%, p<0.05), this difference was not significantly different when compared with preterm-born children (33% vs 23%).

**Table 3:** Skin prick testing for the different groups shown

	POLD-reversible	POLD-fixed	pPRISm	pDysanapsis	Preterm controls	Term
Negative skin prick test (n)	12	7	24	7	76	63
Positive skin prick test (n)	6	2	6	3	23	8
Per cent with positive skin prick test	33%*	22%	20%	30%	23%	11%
Total	18	9	30	10	99	71

*Significant versus T_c_ (single symbol denotes significance level <0.05).

pDysanapsis, dysanapsis of prematurity; POLD, prematurity-associated obstructive lung disease; pPRISm, prematurity-associated PRISm.

### Association of early and current life factors with the four phenotypes

Finally, we used multinomial regression to assess which early and current life factors, including sex, BPD, IUGR, antenatal maternal smoking, deprivation, current BMI and postnatal weight change in z-scores from birth to current weight, were associated with each of abnormal spirometry group with preterm controls as the reference population (table 4). Univariable analyses showed that BPD (OR 3.35; 95% CI 1.43 to 7.83) and IUGR (4.56; 1.89 to 10.96) were associated with the subsequent development of POLD-reversible; and BPD (3.75; 1.43 to 9.83) with the development of POLD-fixed. Weight gain change of z-scores from birth to the current weight (1.37; 1.07 to 1.76) was associated with development of pDysanapsis. The association of BMI z-scores with pPRISm (0.79; 0.62 to 1.01, p=0.064) and IUGR with pDysanapsis (2.13; 0.87 to 5.17, p=0.097) had p values of <0.1. Thus, BPD was associated with the POLD-fixed group, and possibly BMI z-scores with pPRISm. Since the POLD-reversible group was associated with both BPD and IUGR; and the pDysanapsis group with both weight gain and marginally with IUGR (table 4), these factors were included in a multivariable multinomial regression model with outcomes of POLD-reversible and pDysanapsis groups assessed against the preterm controls as the reference population. The results confirmed that POLD-reversible was associated with BPD and IUGR and pDysanapsis group with postnatal weight gain (table 4).

**Table 4:** (A) Univariable and (B) multivariable multinomial regression analyses using early and current life factors to associate with different spirometry phenotypes in the preterm population only

(A)					
Univariable multinomial regression		POLD-reversible	POLD-fixed	pPRISm	pDysanapsis
Sex	Beta (SE)	−0.56 (0.43)	−0.052 (0.48)	0.24 (0.31)	0.073 (0.37)
Female=ref	OR	0.57	0.95	1.27	1.08
	(95% CI)	0.24 to 1.33	0.37 to 2.44	0.70 to 2.30	0.52 to 2.21
	P value	0.19	0.91	0.44	0.84
IUGR	Beta (SE)	1.52 (0.45)	0.77 (0.59)	0.24 (0.44)	0.75 (0.45)
No IUGR=ref	OR	4.56	2.17	1.27	2.13
	(95% CI)	1.89 to 10.96	0.69 to 6.85	0.54 to 2.97	0.87 to 5.17
	P value	0.0007	0.187	0.589	0.097
BPD	Beta (SE)	1.21 (0.43)	1.32 (0.49)	0.420 (0.36)	−0.40 (0.55)
No BPD=ref	OR	3.35	3.75	1.52	0.67
	(95% CI)	1.43 to 7.83	1.43 to 9.83	0.76 to 3.06	0.23 to 1.97
	P value	0.005	0.007	0.24	0.46
WIMD Quintile 2019 (ref=1—highest)					
Fifth—lowest	Beta (SE)	−1.01 (0.75)	0.76 (0.82)	0.063 (0.47)	0.60 (0.68)
	OR	0.37	2.1	1.07	1.83
	(95% CI)	0.085 to 1.58	0.43 to 10.55	0.43 to 2.67	0.48 to 6.97
	P value	0.177	0.354	0.89	0.378
Fourth	Beta (SE)	−0.024 (0.63)	0.2 (0.93)	−0.088 (0.51)	0.31 (0.75)
	OR	0.98	1.22	0.92	1.36
	(95% CI)	0.29 to 3.34	0.20 to 7.51	0.34 to 2.50	0.31 to 5.88
	P value	0.97	0.83	0.863	0.683
Third	Beta (SE)	0.14 (0.63)	0.65 (0.88)	0.36 (0.49)	0.25 (0.78)
	OR	1.15	1.92	1.44	1.28
	(95% CI)	0.34 to 3.94	0.34 to 10.80	0.56 to 3.73	0.28 to 5.92
	P value	0.823	0.46	0.455	0.753
Second	Beta (SE)	−0.32 (0.69)	−0.095 (1.01)	−0.38 (0.57)	1.20 (0.67)
	OR	0.73	0.91	0.68	3.33
	(95% CI)	0.19 to 2.82	0.13 to 6.63	0.23 to 2.06	0.89 to 12.44
	P value	0.645	0.925	0.498	0.073
Antenatal maternal smoking	Beta (SE)	−0.37 (0.75)	−0.053 (0.77)	−0.13 (0.50)	0.42 (0.51)
No smoking=ref	OR	0.69	0.95	0.88	1.52
	(95% CI)	0.16 to 3.02	0.21 to 4.25	0.33 to 2.34	0.55 to 4.15
	P value	0.622	0.944	0.8	0.417
Weight change from birth to current weight	Beta (SE)	0.10 (0.14)	−0.004 (0.16)	0.08 (0.10)	0.32 (0.13)
	OR	1.1	1	1.08	1.37
	(95% CI)	0.83 to 1.46	0.73 to 1.36	0.88 to 1.32	1.07 to 1.76
	P value	0.488	0.982	0.452	0.013
BMI z-score	Beta (SE)	−0.24 (0.17)	−0.16 (0.20)	−0.23 (0.13)	0.21 (0.15)
	OR	0.79	0.85	0.79	1.23
	(95% CI)	0.56 to 1.11	0.58 to 1.26	0.62 to 1.01	0.92 to 1.64
	P value	0.17	0.42	0.064	0.17

(B)			
Multivariable multinomial regression		POLD-reversible	pDysanapsis
BPD	Beta (SE)	1.09 (0.44)	−0.40 (0.55)
No BPD=ref	OR	3	0.67
	(95% CI)	1.24 to 7.07	0.23 to 1.99
	P value	0.015	0.471
IUGR	Beta (SE)	1.56 (0.52)	0.31 (0.51)
No IUGR=ref	OR	4.74	1.37
	(95% CI)	1.70 to 13.22	0.51 to 3.70
	P value	0.003	0.537
Weight change from birth to current weight	Beta (SE)	−0.089 (0.16)	0.293 (0.145)
	OR	0.91	1.34
	(95% CI)	0.67 to 1.26	1.01 to 1.79
	P value	0.58	0.043

Reference is the preterm control population.

BMI, body mass index; BPD, bronchopulmonary dysplasia; IUGR, intrauterine growth restriction; pDysanapsis, dysanapsis of prematurity; POLD, prematurity-associated obstructive lung disease; pPRISm, prematurity-associated PRISm; WIMD, Welsh Index of Multiple Deprivation.

## Discussion

As expected, we identified groups of children with fixed and variable airflow obstruction including, and, contrary to previous literature,^10^ a subgroup of children with increased FE_NO_. We have also demonstrated that dysanaptic airway growth and PRISm are part of the spectrum of lung abnormalities following preterm birth. Thus, spirometry identified four phenotypes of PLD, representing 22.6% of the preterm population compared with 9.2% in the term group. We have also reported associations of each phenotype with presence of early or current life factors with BPD, IUGR, BMI and weight gain between birth and current weight being most relevant.

The study of respiratory outcomes after preterm birth has largely focused on those who survived the diagnosis of BPD in infancy. Increasingly, it is recognised that many preterm-born survivors of BPD do not develop respiratory disease in later life.^8^ In contrast, many born at later gestations especially at 33–34 weeks’ gestation are also at risk of later lung function decrements.^8^ Furthermore, it is unclear which groups are associated with bronchodilator responses and with FE_NO_, which has not shown to be increased in those who had BPD in infancy.^10^

Several studies show that survivors of prematurity especially those who had BPD in infancy respond to single doses of bronchodilators but long-term evaluation of bronchodilators is limited.^9^ We identified two obstructive airway groups, one with low FEV_1_ (POLD groups) and a second in which FEV_1_ was ≥LLN and FEV_1_/FVC ratio<LLN (pDysanapsis) as suggested by the ATS/ERS guidelines.^15^ We subclassified the POLD group into those who did and did not respond to bronchodilators. In addition, a subgroup of the PRISm patients (13%) was also bronchodilator responsive. Bronchodilator responsiveness is an important group as it is reasonable to speculate that these children, especially those whose FEV_1_ improved to greater than LLN, should benefit from inhaler treatment with combined ICS and LABA as we have recently reported.^11^ It should be noted that the evidence for corticosteroid responsive airway eosinophilia is not present in most POLD children, but there are insufficient safety data for using LABA alone in this group. In our previous meta-analyses, FE_NO_ was not different between term and preterm groups including those who had BPD in infancy.^10^ Only 45% of the POLD-reversible group had an increased FE_NO_ thus mechanisms other than atopy and airway eosinophilia are important in this group. In support of this, only 33% had a positive skin test. Inevitably, a number of children born preterm will have coincident atopic asthma.^1^ Preterm-born infants who die from BPD have been shown to have smooth muscle extending much further distally in the airways at autopsy than non-BPD and term-born controls.^24 25^ It is likely that the smooth muscle remains responsive to bronchodilators at least in a proportion of the population with POLD. On multivariable modelling, both BPD and IUGR were strongly associated with the development of POLD-reversible disease. Historically, focus for long-term respiratory outcomes has been on preterm-born children with BPD.^2 26^ The BPD group with low lung function were more likely to develop POLD rather than pPRISm, when compared with non-BPD preterm-born children with low lung function. This may be due to the greater number of interventions, including respiratory support and prolonged supplemental oxygen, that these children will have been exposed to in the neonatal period. While this is speculative, some support is provided by the above mentioned increased distal airway smooth muscle extension that has been observed in infants who die from BPD when compared with non-BPD controls.^24 25^ Additional factors such as maternal antenatal smoking and postnatal exposure to smoking and pollution as well as antenatal and postnatal use of corticosteroids in infancy and childhood could potentially lead to, and/or potentially modify, the eventual phenotype. However, prospective studies will be required, especially to evaluate if treatment can modulate the lung disease and potentially modulate the phenotype.

The POLD-fixed group may potentially have progressed from a reversible to a fixed phenotype, but this is speculative. This lack of reversibility may be explained by the distal extension of smooth muscle hypertrophy remodelling into myofibroblasts which is unlikely to respond to inhaled therapy. Alternatively, failure of normal airway development may be a factor. This group did not have increased FE_NO_ and were no more likely to be skin prick test positive than the preterm group (22% vs 23%). Interestingly, the POLD-fixed group was associated with BPD in multinomial regression modelling but not with IUGR thus again suggesting that early intervention with mechanical ventilation, supplemental oxygen therapy, etc in the neonatal period is likely to result in obstructive airway disease possible due to lung injury to the parenchyma and to the airways.

Only 41% of the pDysanapsis group responded to bronchodilators, and 31% had FE_NO_ over 35 ppb. Positive skin prick tests were noted in 30% against 11% and 23% in the term and preterm groups. Early weight gain in infancy in both term and preterm born children has been shown to result in dysanapsis.^27 28^ On univariable analyses, weight gain between birth and current weight was significantly (p=0.013) and IUGR was weakly (p=0.097) associated with the pDysanapsis group with only the former remaining significantly associated on multivariable regression analyses. This group was not associated with BPD. The pDysanapsis group had increased respiratory symptoms, doctor diagnosed asthma and inhaler use. Of concern is that this group of children is likely to have similar worse outcomes as observed for asthma in school age children and adults.^14^

Although it is tempting to dismiss the group with %FEV_1_<LLN and FEV_1_/FVC ratio≥LLN as children with small lungs, longitudinal studies into late adulthood are increasingly showing that low lung function trajectories lead to premature development of COPD even without accelerated decline.^29–31^ Furthermore, PRISm phenotype is increasingly being recognised and associated with increased cardiorespiratory morbidity and all-cause mortality.^12 13^ Most studies have been of adults at middle age or beyond with little described in children. Since prematurity is associated with early life decreases in spirometry, it is very likely that this group of subjects will progress to develop a prematurity-associated PRISm phenotype. As with the observations in adults, we noted no increase in FE_NO_ and a subgroup of 13% responded to bronchodilators. On univariable multinomial regression, there was no association between weight gain between birth and current age but there was a near statistically significant negative association with BMI (beta=−0.23, p=0.064). There was no effect of sex or BPD.

We have shown that there are three different phenotypes that may respond to bronchodilators, some of which are associated with increased FE_NO_ and atopy. We have also recently showed that preterm-born children with POLD have decreased exercise capacity as well as increased functional residual capacity (FRC) and residual volume (RV) when compared with term-born controls.^32^ In contrast, those with pPRISm had intermediate decreases in exercise capacity measures but had decreased FRC and RV. Some but not all small studies thus far have shown an increase in neutrophils^33^ or oxidant injury^34^ but evidence for airway eosinophilic inflammation in this group is lacking. Credence is given to an active role for the increased FE_NO_ in the obstructive airway phenotypes as we noted decreased FE_NO_ in a recent randomised controlled trial in both arms of ICS alone and when used in combination with LABA. Clinically inhaled corticosteroids non-significantly improved %FEV_1_ by ~7% but the combination of ICS and LABA resulted in >14% significant improvement. The current data suggest that such a trial of therapy is not unreasonable for the POLD-obstructive and pDysanapsis groups and a supervised prolonged trial may be of benefit to those who do not respond to single doses of bronchodilators, especially if any inflammatory component contributes to the underlying airway disease. Whether the pPRISm group and the POLD-fixed group respond to prolonged therapy is speculative but a similar trial of supervised prolonged combined inhaled treatment is not unreasonable.

There are a number of strengths and weaknesses in this study. The main strength is that we have studied the largest group of preterm-born children with standardised objective assessments that has permitted the classification into different phenotypes. Although we have recently reported association of lung volumes with POLD and pPRISm,^32^ the majority of the children, especially those preterm-born children in the pPRISm group, did not have their lung volumes assessed to enable classification into restrictive or mixed lung disease. While we did not have total lung volumes for all the children, our primary aim was to identify specific phenotypes that can be identified in the general outpatient clinic where spirometry and FE_NO_ measurements are often routine. Although some of the data may be subject to recall bias, we have based the classification into phenotypes by using objective measures which are not influenced by recall bias. In addition, although we used the definition for dysanapsis suggested by the ATS/ERS guidelines, we are aware that there are a number of related definitions^14 35^ and agree further work is required to identify an ideal definition which is applicable to both children and adults.

In summary, we have identified four phenotypes of PLD which have differential associations with bronchodilators, FE_NO_ and with early life factors. These phenotypes especially the POLD group were recently shown to be associated with imaging abnormalities.^36^ The burden of respiratory disease is significant as 23% of the preterm population, compared with 9.2% of term-born children, was associated with at least one of these four phenotypes. By studying the mechanisms that underly these endotypes, we hope, specifically targeted therapies to prevent or improve respiratory outcomes of preterm-born children can be developed.

## Data Availability

Data from the RHiNO study are available to research collaborators subject to confidentiality and non-disclosure agreements. Contact Professor Sailesh Kotecha (kotechas@cardiff.ac.uk) for any data requests.
