# Supplementary material for: Characterising airway obstructive, dysanaptic and PRISm phenotypes of prematurity-associated lung disease
Source: Thorax. 2023 Feb 1;78(9):895–903. doi: 10.1136/thorax-2022-219301 (PMC10447414; doi:10.1136/thorax-2022-219301)
Supplement: Supplementary data [file thorax-2022-219301supp001.pdf]

Characterising Airway Obstructive, Dysanaptic and PRISm Phenotypes of Prematurity-Associated Lung Disease – Online Supplement

[1]Michael Cousins*, [1]Kylie Hart*,[1]Sarah J Kotecha, [2]A John Henderson**, [1]W John Watkins, [3]Andrew Bush, [1]Sailesh Kotecha

(*Joint 1st authors)

**This publication is dedicated to our expert collaborator, valued mentor, and very dear late friend.

[1]Department of Child Health, Cardiff University School of Medicine, Cardiff, United Kingdom

[2] MRC Integrative Epidemiology Unit, Population Health Sciences, Bristol Medical School, University of Bristol, Bristol, United Kingdom.

[3]Centre for Paediatrics and Child Health, Imperial College of Medicine, London, United Kingdom

Corresponding Author: Professor Sailesh Kotecha

Department of Child Health

Cardiff University School of Medicine

Heath Park

Cardiff CF14 4XN

United Kingdom

Email: KotechaS@cardiff.ac.uk

Cousins M, *et al. Thorax* 2023; 78:895–903. doi: 10.1136/thorax-2022-219301

Methods

Clinical and Physiological Assessments

All children underwent assessment with physical examination, height, and weight, along with fractional exhaled nitric oxide (FE_{NO}) (Circassia, Oxford, UK) and spirometry (Microloop, CareFusion, Wokingham, UK) testing, performed by two trained paediatric research nurses. Reversibility testing was performed with administration of a bronchodilator (4 x 100 mcg puffs of salbutamol administered via a spacer device) with repeat spirometry measurements obtained after 15 minutes. Bronchodilator reversibility was defined by an increase of ≥10 percent predicted forced expired volume in one second, $\%FEV_1$. Spirometry was performed and quality controlled as per the American Thoracic Society/ European Respiratory Society guidelines [1], and normalised against GLI reference values[2]. Any respiratory medications were withheld prior to their assessments (short and long acting $beta_2$ agonists for 8 and 48 hours respectively; inhaled corticosteroids for 24 hours; and leukotriene receptor antagonists for 48 hours), and children were free of respiratory infections for at least three weeks prior to testing.

Skin prick testing

Skin prick testing was performed using Multi-Test PC lancets (Lincoln Diagnostics, USA). A Dipwell Tray (Lincoln Diagnostics, USA) was pre-prepared with the following allergens: cat dander; dermatophagoides pterynyssinus; grass mix; dog dander; aspergillus fumigatus; and cladosporium herbarum; as well as a positive histamine control and a negative control (Immunotek, Spain). The procedure was explained to the child and their forearm was cleaned gently with water, after ensuring the skin was free from eczema or any similar skin conditions. The Multi-Test PC lancet was inserted into the Dipwell Tray ensuring all touch-posts were coated with allergen solution. The lancet was slowly removed from the tray, and gently applied to the skin. Following one second of gentle pressure, the lancet was pressed firmly onto the skin with gentle rotation of the lancet device up and down and side to side before removal. Successful application left the imprints of the touch posts on the skin. Any

Cousins M, *et al. Thorax* 2023; 78:895–903. doi: 10.1136/thorax-2022-219301

excess allergen fluid on the skin was gently removed with tissue paper ensuring no cross-contamination of sites. A timer was set for 15 minutes. Children were encouraged not to scratch if the arm got itchy. After 15 minutes the arm was inspected for any wheals that developed; the raised aspects of the wheals were drawn around with pen and tape was used to lift the pen mark and stuck to a data sheet. A ruler was then used to measure the widest diameter of any of the wheals. A test was deemed positive if the wheal was greater than 3 mm, along with a positive histamine control test.

References

1. Miller MR, Hankinson J, Brusasco V, et al. Standardisation of spirometry. *Eur Respir J*. 2005;26(2):319–38. doi:10.1183/09031936.05.00034805.
2. Quanjer PH, Stanojevic S, Cole TJ, et al. Multi-ethnic reference values for spirometry for the 3-95-yr age range: the global lung function 2012 equations. *Eur Respir J*. 2012;40(6):1324–43. doi:10.1183/09031936.00080312.

Cousins M, *et al. Thorax* 2023; 78:895–903. doi: 10.1136/thorax-2022-219301
